# Characterization of carbapenem-resistant *Acinetobacter baumannii* isolates in a Chinese teaching hospital

**DOI:** 10.3389/fmicb.2015.00910

**Published:** 2015-09-01

**Authors:** Yaowen Chang, Guangxin Luan, Ying Xu, Yanhong Wang, Min Shen, Chi Zhang, Wei Zheng, Jinwei Huang, Jingni Yang, Xu Jia, Baodong Ling

**Affiliations:** ^1^Small Molecule Drugs Sichuan Key Laboratory, Institute of Materia Medica, Chengdu Medical CollegeChengdu, China; ^2^Non-coding RNA and Drug Discovery Laboratory, Chengdu Medical CollegeChengdu, China; ^3^Clinical Laboratory, The First Affiliated Hospital, Chengdu Medical CollegeChengdu, China; ^4^Institute of Antibiotics, The Fifth Affiliated Hospital, Wenzhou Medical UniversityLishui, China

**Keywords:** *Acinetobacter baumannii*, carbapenem resistance, OXA-23, plasmid, CC92

## Abstract

Carbapenem-resistant *Acinetobacter baumannii* (CRAB) presents a serious therapeutic and infection control challenge. In this study, we investigated the epidemiological and molecular differences of CRAB and the threatening factors for contributing to increased CRAB infections at a hospital in western China. A total of 110 clinical isolates of *A. baumannii*, collected in a recent 2-year period, were tested for carbapenem antibiotic susceptibility, followed by a molecular analysis of carbapenemase genes. Genetic relatedness of the isolates was characterized by multilocus sequence typing. Sixty-seven of the 110 isolates (60.9%) were resistant to carbapenems, 80.60% (54/67) of which carried the *bla*_OXA-23_ gene. Most of these CRAB isolates (77.62%) were classified as clone complex 92 (CC92), and sequence type (ST) 92 was the most prevalent STs, followed by ST195, ST136, ST843, and ST75. One CRAB isolate of ST195 harbored plasmid pAB52 from a Chinese patient without travel history. This plasmid contains toxin–antitoxin elements related to adaptation for growth, which might have emerged as a common vehicle indirectly mediating the spread of OXA-23 in CRAB. Thus, CC92 *A. baumannii* carrying OXA-23 is a major drug-resistant strain spreading in China. Our findings indicate that rational application of antibiotics is indispensable for minimizing widespread of drug resistance.

## Introduction

*Acintobacter baumannii* is an opportunistic Gram-negative pathogen, which has recently been successfully spreading worldwide nosocomial infections and causing outbreaks of hospital-acquired infections, primarily due to its prominent ability to acquire antibiotic resistance (Peleg et al., 2008; Kempf and Rolain, 2012). *A. baumannii* is developing into multidrug resistant (MDR), extensively drug resistant (XDR), and pandrug resistant (PDR) bacteria, and its adaptation to the environment with drug resistance has previously been reported (Durante-Mangoni and Zarrilli, 2011). Carbapenems were regarded as the most powerful antibiotics because of its extremely effective antibacterial activity and low toxicity, but the emergence of carbapenem resistance in *A. baumannii* has become a global concern recently (Peleg et al., 2008; Tiwari et al., 2012a,b). Resistance of *A. baumannii* to expanded-spectrum cephalosporins and carbapenems is rapidly growing over the years (Bradford, 2001; Peleg et al., 2008). For example, surveillance by the CHINET project in China revealed that the rate of resistance of *A. baumannii* to the carbapenem, such as imipenem, doubled from 30.1% in 2006 to 62.8% in 2013 (Wang, 2008; Fupin et al., 2014).

Various types of β-lactam antibiotics, for example carbapenems, contain β-lactam rings in their structures and can be inactivated by β-lactamase enzymes. The β-lactamases are classified into four different molecular groups, Ambler classes A through D, according to amino acid sequence identities (Bush et al., 1995). Class A, C, and D (OXA enzymes) of β-lactamases contain a catalytically active serine residue that cleaves the lactam ring of antibiotics (Bush et al., 1995; Tiwari and Moganty, 2014). Class B of β-lactamases is a metallo-enzyme that requires zinc for their catalytic activity, and therefore, has a completely different mechanism for enzyme activity (Tiwari and Moganty, 2013). Carbapenems, such as imipenem and meropenem, have an exceedingly broad spectrum of activity and are able to resist hydrolysis by most of the β-lactamases, including extended-spectrum and derepressed class C chromosomal AmpC β-lactamases (Bush et al., 1995). However, most of the metallo-β-lactamases and a number of class A and D β-lactamases are able to hydrolyse broad spectrum carbapenems, such as imipenem and meropenem (Walther-Rasmussen and Hoiby, 2006; Evans and Amyes, 2014). Thus, outbreaks of OXA-23-producing *A. baumannii* have been reported from various regions of the world (Carvalho et al., 2009). It is generally believed that OXA-23 is responsible for carbapenem antibiotic resistance. Previously, Liu et al. (2015) reported the dissemination of MDR OXA-23-producing *A. baumannii* clones throughout multiple cities in China, but little is known about the molecular mechanisms of resistance to carbapenems in western China.

The multilocus sequence typing (MLST) has been widely applied in genotyping of bacteria, including *A. baumannii* (Bartual et al., 2005). Molecular epidemiological research of *A. baumannii* indicates that CC92 has played an important role in nosocomial infection outbreak and spread nationwide (Runnegar et al., 2010).

This study aimed to report the dissemination of *A. baumannii* harboring carbapenemase genes throughout a university hospital in western China, then identify the risk factors for carbapenem-resistant *Acinetobacter baumannii* (CRAB) infections, and finally perform a comprehensive evaluation and comparison of their genetic diversity.

## Materials and Methods

### Bacteria Isolates

A total of 110 consecutive and non-duplicated *A. baumannii* clinical isolates were collected from different departments [intensive care unit (ICU), gastroenterology, respiratory, neurosurgery and other wards] at the First Affiliated Hospital of Chengdu Medical College, Chengdu, Sichuan, China from 2012 to 2013. Isolates were identified by standard laboratory methods and ATB New (bioMérieux, France). *A. baumannii* was further verified when two PCR products were yielded as reported: a 425-bp internal control amplicon corresponding to the *recA* gene of *Acinetobacter* spp. and the 208-bp fragment of the 16S rRNA (Chiang et al., 2011) intergenic spacer region of *A. baumannii* (**Table 1**). All strains were stored at –80°C, and bacteria were grown on tryptose agar or Mueller–Hinton broth or agar (Oxoid, England).

**Table 1:** Primers used in this study.

Locus	Primer	Oligonucleotides (5′→ 3′)	Expected size (bp)	Source
16S	F	CAT TAT CAC GGT AAT TAG TG	208	Chiang et al. (2011)
	R	AGA GCA CTG TGC ACT TAA G		
RecA	F	CCT GAA TCT TCT GGT AAA AC	425	Chiang et al. (2011)
	R	GTT TCT GGG CTG CCA AAC ATT AC		
KPC	F	GCT CAG GCG CAA CTG TAA GT	823	Li et al. (2014)
	R	GTC CAG ACG GAA CGT GGT AT		
IMP	F	CTA CCG CAG AGT CTT TG	587	Valenzuela et al. (2007)
	R	AAC CAG TTT TGC CTT ACC AT		
VIM-1	F	AGT GGT GAG TAT CCG ACA G	261	Tsakris et al. (2000)
	R	ATG AAA GTG CGT GGA GAC		
17SIM	F	TAC AAG GGA TTC GGC ATC G	570	Ellington et al. (2007)
	R	TAA TGG CCT GTT CCC ATG TG		
NDM-1	F	TCT CGA CAT GCC GGG TTT CGG	475	Yong et al. (2009)
	R	ACC GAG ATT GCC GAG CGA CTT		
AmpC	F	ACT TAC TTC AAC TCG CGA CG	663	Bou and Martinez-Beltran (2000)
	R	TAA ACA CCA CAT ATG TTC CG		
OXA-23	F	GAT CGG ATT GGA GAA CCA GA	501	Woodford et al. (2006)
	R	ATT TCT GAC CGC ATT TCC AT		
OXA-24	F	CAA GAG CTT GCA AGA CGG ACT	420	Woodford et al. (2006)
	R	TCC AAG ATT TTC TAG CRA CTT ATA		
OXA-51	F	TAA TGC TTT GAT CGG CCT TG	353	Fu et al. (2010)
	R	TGG ATT GCA CTT CAT CTT GG		
OXA-58	F	TCG ATC AGA ATG TTC AAG CGC	530	Netsvyetayeva et al. (2011)
	R	ACG ATT CTC CCC TCT GCG C		
OXA-235	F	TTG TTG CCT TTA CTT AGT TGC	831	Higgins et al. (2013)
	R	CAA AAT TTT AAG ACG GAT CG		

### Minimal Inhibitory Concentration (MIC)

The minimal inhibitory concentration (MIC) of carbapenems including imipenem and meropenem for *A. baumannii* were determined by the agar dilution method as previously described in the guidelines from the Clinical and Laboratory Standards Institute (CLSI, 2014). *Escherichia coli* ATCC25922 and *A. baumannii* ATCC19606 were used as quality control strain. The results were interpreted according to the CLSI guidelines (CLSI, 2014), i.e., CRAB was defined as an *A. baumannii* isolate that was resistant to both imipenem and meropenem (i.e., ≥8 μg/ml as resistant), whereas carbapenem-susceptible *A. baumannii* (CSAB) possessed carbapenem MIC of ≤2 μg/ml and carbapenem-intermediate *A. baumannii* (CIAB) has MIC of 4 μg/ml).

### PCR Experiments

The genes encoding carbapenemases class A [e.g., *Klebsiella pneumoniae* carbapenemase gene, *bla*_KPC_ (Li et al., 2014)], class B [e.g., the metallo-β-lactamases, *bla*_IMP_ (Valenzuela et al., 2007), *bla*_V IM-1_ (Tsakris et al., 2000), *bla*_SIM_ (Ellington et al., 2007), and *bla*_NDM-1_ (Yong et al., 2009)], class C [e.g., *bla*_AmpC_ (Bou and Martinez-Beltran, 2000)], and class D [e.g., *bla*_OXA-23_ (Woodford et al., 2006), *bla*_OXA-24_ (Woodford et al., 2006), *bla*_OXA-51_ (Fu et al., 2010), *bla*_OXA-58_ (Netsvyetayeva et al., 2011), and *bla*_OXA-235_ (Higgins et al., 2013)] were investigated by polymerase chain reaction (PCR).

To prepare DNA templates, each isolate was grown on tryptose agar (Oxoid, England) plate overnight. Two to three colonies were suspended in 100 μl of sterile distilled water in a 1.5-ml Eppendorf tube, and the suspension was heated at 100°C for 15 min, followed by centrifugation at 12,000 *g* for 10 min to pellet the debris. The resultant supernatant was used as the DNA template in the PCRs, which were carried out in a 50-μl volume containing 0.2 mM each deoxynucleotide, 0.5 μM each primer, 1.25 U of Taq polymerase, and 5 μl of 10× buffer (Thermo, China). Primers (**Table 1**) were synthesized by Sangon Company (Sangon, China). Reaction conditions of PCR were 94°C for 5 min and 30 cycles of 94°C for 30 s, 55°C for 30 s (57°C for *bla*_AmpC_ amplification), and 72°C for 30 s, followed by a final extension at 72°C for 5 min.

### Multilocus Sequence Typing

MLST was used to describe the genetic backgrounds of CRAB and CSAB in all clinical isolates (Diancourt et al., 2010; Hamouda et al., 2010; Pournaras et al., 2014). eBURST was performed to cluster sequence types (STs) into clonal complexes (CCs) and infer evolutionary descent. MLST was carried out as described by Bartual et al. (2005) for all isolates. In brief, internal fragments of seven housekeeping genes, *gltA, gyrB, gdhB, recA, cpn60, gpi*, and *rpoD*, were amplified, purified, and sequenced (Fu et al., 2010; Ruan et al., 2013). And eBURST (version 3, http://eburst.mlst.net/) was used to assign STs to CCs and define the genetic relatedness of STs with the most stringent definition of the groups by sharing the same alleles at ≥6 of 7 loci (Feil et al., 2004).

### Plasmid Conjugation, Extraction, Sequencing, and Analysis

Plasmid conjugations were performed by using meropenem-resistant *A. baumannii* as donors and an azide-resistant *E. coli* J53 as the recipient (MIC > 200 μg/ml; Bogaerts et al., 2006). Matings were performed as described previously (Wang et al., 2003), and transconjugants were recovered on L-agar plates containing meropenem (8 μg/ml) and azide (150 μg/ml).

Plasmid DNA was extracted using a Plasmid Midi Kit (Omega, USA) according to the manufacturer’s instructions. The plasmid was cut by EcoRI, then the fragments were ligated to the vector pET21a. The complete sequencing work was performed by the ABI-3730 XL system (Biosune Biotechnology Company, Shanghai, China) and sequences were assembled by Life Technologies (BigDye V3.1, four fluorescence reagent kit). The sequenced plasmid was annotated by the RAST server (Aziz et al., 2008), then all of the predicted proteins were further compared against the NCBI non-redundant protein database using the BLASTP program. In addition, the DNAMAN software (Lynnon Corporation, USA) was used to generate a circular map of plasmid pAB52.

### Nucleotide Sequence Accession Numbers

The complete nucleotide sequences (Supplementary Table S1) of plasmid pAB52 in this study were submitted to GenBank with assigned accession number KR030046.

## Results

### Characterization of Clinical Isolates

According to our previous study, 67 of the total 110 *A. baumannii* isolates exhibited resistance to carbapenems, imipenem and meropenem, with MIC values of ≥8 μg/ml. These CRAB isolates were from different types of specimens including sputum (*n* = 49), throat swabs (*n* = 5), blood (*n* = 1), douche (*n* = 4), and others (*n* = 8). Epidemiological analysis of the 67 patients with CRAB revealed that 27 were ≥70 years old, 15 were between 60 and 70 years old, 24 were between 21 and 60 years old, and one was ≤20 years old. Among 40 males (59.7%) and 27 females (40.3%), 24 isolates were collected from the ICU, and 17 isolates were collected from the respiratory department. Clinical characteristics of the 67 patients with CRAB are summarized in **Table 2**.

**Table 2:** Clinical characteristics of 67 patients with Carbapenem-resistant *Acinetobacter baumannii* (CRAB).

Gender	Total number	Source	Total number	Department	Total number
Male	40 (59.70%)	Blood	1 (1.49%)	Intensive care unit	24 (35.82%)
Female	27 (40.30%)	Sputum	49 (73.13%)	Respiratory	17 (25.37%)
**Age (years)**	**Total number**	Swab	5 (7.46%)	Neurosurgery	9 (13.43%)
0–20	1	Secretion	5 (7.46%)	Infectious diseases department	2 (2.99%)
21–50	11	Douche	4 (5.98%)	Other wards	15 (22.39%)
51–60	13	Cerebrospinal fluid	2 (2.99%)		
70–more	27	Other	2 (2.99%)		

### Expression of Carbapenemase Genes in *A. baumannii* Isolates

All isolates were screened for the presence of β-lactamases genes (**Table 3**). In the 67 CRAB isolates, all of them were positive to *recA*, 16S rRNA conservative region and *bla*_OXA-51_. The major carbapenemase gene *bla*_OXA-23_ was detected in 80.60% of all isolates. In addition, 65 isolates (97.10%) were found to carry *bla*_AmpC_. Other β–lactamase genes including *bla*_KPC_, *bla*_IMP_, *bla*_V IM-1_, *bla*_SIM_, *bla*_NDM-1_, *bla*_OXA-24_, *bla*_OXA-58_, and *bla*_OXA-235_ were undetectable in all isolates. Thus, the expression of *bla*_OXA-23_ is still the dominant carbapenem resistance mechanism in *A. baumannii* among the isolates investigated aside from the inherent *bla*_OXA-51_ gene. One susceptible isolate carried *bla*_OXA-72_.

**Table 3:** Positive rates of β-lactamase genes in CRAB.

No. of strains	Rate of gene (%)										
	KPC	IMP	VIM-1	SIM	NDM-1	AmpC	OXA-23	OXA-24	OXA-51	OXA-58	OXA-235
67	0	0	0	0	0	97.10	80.60	0	100	0	0

### Genetic Analysis of STs

**FIGURE 1:**
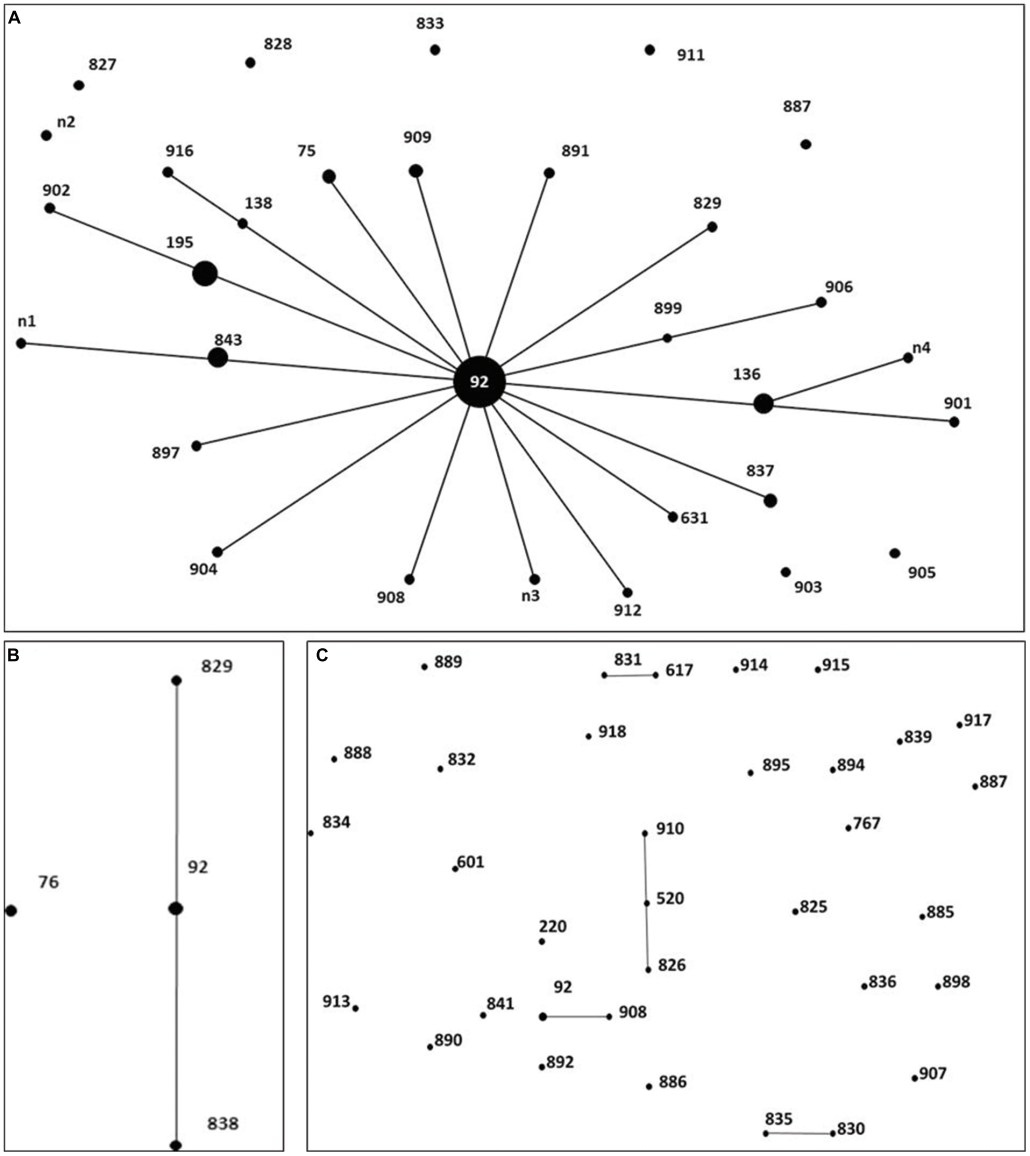
**Distribution of genes in 67 Carbapenem-resistant *Acinetobacter baumannii* (CRAB), 8 carbapenem-intermediate *A. baumannii* (CIAB), and 35 carbapenem-susceptible *A. baumannii* (CSAB).** Minimum spanning tree analysis by eBURST algorithm of CRAB **(A)**, CIAB **(B)** and CSAB **(C)** isolates based on MLST data. Each circle represents a specific sequence type (ST). The size of each circle homologizes to a different number of isolates, with larger sizes representing higher frequency of occurrence. The solid lines connecting the circles indicate the relationship between different STs.

To investigate whether the isolates were genetically related, MLST was performed to characterize the CRAB, CIAB and CSAB (**Figure 1**). Each ST was represented by a dot that was proportionally sized to the number of *A. baumannii* containing that ST. The MLST analysis revealed a total of 32 different STs in 67 CRAB, including 6 existing STs and 26 novel STs. 77.62% of the 67 CRAB isolates were classified to CC92, ST92 and its 4 novel single-locus variants (SLVs), ST195, ST136, ST843, and ST75, were the predominant STs, found in 34.33, 7.46, 5.97, 4.48, and 2.99% of isolates, respectively. Among these five predominant STs, 82.35% of carbapenem-resistant isolates carried the *bla*_OXA-23_ gene, in which ST92 is one of the most widespread STs across the world and belongs to the CC92 in *A. baumannii* MLST databases (Wang et al., 2013). Seventeen of the 32 CRAB STs belong to CC92 (**Figure 1A**). In this study, the number of polymorphic sites in *gpi* (*n* = 8), *rpoD* (*n* = 4), *cpn60* (*n* = 3), *recA* (*n* = 2), and the other three loci (*gdhB, gyrB*, and *gltA*) indicated that all were the same in CC92. Seven of the eight CIAB were CC92 (**Figure 1B**), but only 3 of the 35 CSAB were CC92 (**Figure 1C**). All these suggested that CC92 represents the most widely distributed carbapenem-resistant CC identified in the hospital.

### Plasmid Analysis

To detect the nosocomial infection mediated by *A. baumannii* plasmid DNA, a plasmid conjugation assay was performed. However, none of the carbapenem-resistant isolates transferred plasmids from *A. baumannii* isolates to *E. coli* J53 successfully. This could be due to multiple reasons. Carbapenem resistance genes were located in the genome, and plasmids were not conjugative or unable to replicate in the *E. coli* host. Of note, with no. 52 CRAB isolate (which harbored pAB52 plasmid) as the donor strain, a few colonies with azide resistance and displaying a similar morphology as the *E. coli* J53 recipient were obtained from the conjugation. Yet, these colonies were found to be arisen from *A. baumannii* based on the amplification and sequence analysis of 16S rRNA and seven housekeeping genes *gltA, gyrB, gdhB, recA, cpn60, gpi*, and *rpoD*. Likely, the generation of azide-resistant *A. baumannii* was a result of spontaneous mutation(s). In addition, plasmid extraction assay confirmed no plasmid-containing *E. coli* J53 colonies, indicating that pAB52 could not be transferred from *A. baumannii* to *E. coli* J53.

**FIGURE 2:**
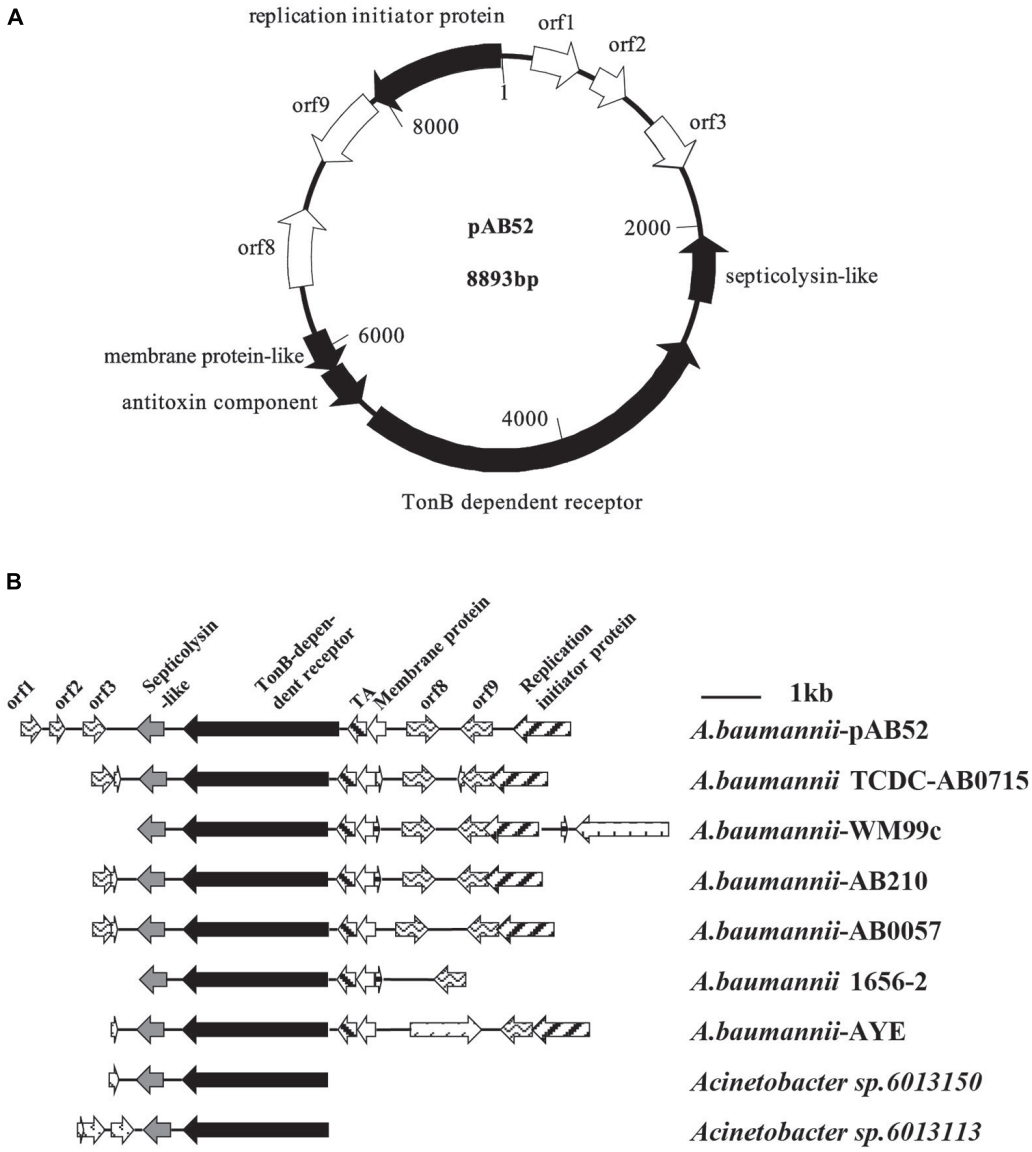
**(A)** Diagram of plasmid pAB52 (GenBank accession number KR03004). The open reading frames (ORFs), represented by arrows pointing in the direction of transcription, are white, except for that for the reported genes, which is black. **(B)** Comparative analysis of the genetic environment of the TonB-dependent receptor in plasmid DNA by RAST software. Regions with 100% nucleotide sequence identity or similar biological function are filled with the same pattern.

RAST analysis showed that the plasmid pAB52 was 8,893 bp in size and contained 10 open reading frames (ORFs; **Figure 2A**), with an average G+C content of 34.5%. Three genes with a putative role in virulence were detected in pAB52: a septicolysin-like gene coding for a pore-forming toxin (Rosado et al., 2008; Gan et al., 2012), a TonB-dependent receptor gene coding for an outer membrane protein involved in iron uptake and virulence (Acosta et al., 2011; Gan et al., 2012), and a toxin–antitoxin (TA) gene coding for is a functional TA system and that its toxin, SplT (Gan et al., 2012; Jurenaite et al., 2013), inhibits translation (**Figure 2A**). One ORF (orf 9) codes for proteins that share similarity to hypothetical proteins encoded by plasmid genes found in other bacteria (Gan et al., 2012), while the predicted products of three others (orf 1, orf 2, and orf 3) do not match any known sequences. orf 9, which is adjacent to the replication initiation protein, is similar to replication protein of other genomes (**Figure 2B**). Therefore, it might contribute to a replication ability. Further analysis indicated that this plasmid shared most nucleotide identity with previously described isolates of *A. baumannii*, WM99C, TCDC-AB0715, AB210, AB0057, 1656-2, AYE, UMB001, and ATCC17978, *Acinetobacter sp.* 6013150 and 6013113 (**Figure 2B**).

## Discussion

Clinical characterization of 67 patients with CRAB indicated that compromised immunity (e.g., elderly patients suffering from respiratory failure, encephalorrhagia, extensive burns, and chronic obstructive pulmonary disease) increases the risk of *A. baumannii* infection. In addition, males appear to have a greater risk of infection than females.

Surveillance by the CHINET project demonstrated that the *A. baumannii* rate of imipenem resistance increased over twofold between 2006 and 2013 to 62.8% in China (Fupin et al., 2014). In contrast, the resistance rate of *Acinetobacter* spp. to cefoperazone/sulbactam was only 36.4% (Fupin et al., 2014), far below the rates of imipenem resistance. Mirroring the national trend, the *A. baumannii* resistance rate of imipenem and cefoperazone/sulbactamat in our hospital was 60.9 and 14.75%, respectively (Ying et al., 2013).

We observed that OXA-23 was also the major carbapenemase mechanism responsible for the resistance phenotype (**Table 3**), as the gene was expressed in most CRAB isolates. This result is consistent with previous reports in which the CRAB is often caused due to the expression of OXA enzymes, particularly OXA-23 (Fu et al., 2010; Merino et al., 2014; Zowawi et al., 2015).

In our experiments, we unexpectedly found one particular carbapenem-susceptible isolate, which belonged to ST220 and carried the *bla*_OXA-72_ gene, a member of the *bla*_OXA-24_ subgroup, but this isolate did not carry any other resistance genes. This is the first time that *bla*_OXA-24_ has been detected in western China. Of note, this isolate was not only susceptible to meropenem and imipenem, but also susceptible to other β-lactams, aminoglycosides, tetracyclines, fluoroquinolone, rifampicin, and trimethoprim–sulfamethoxazole (data not shown). Our results are in contrast with previous reports that nosocomial infections were triggered by CRAB-carrying plasmids encoding OXA-72 carbapenemase via plasmid DNA from Lithuanian (Povilonis et al., 2013) and Taiwan (Lu et al., 2009). There are several possible reasons for the different results. For instance, the resistance genes may not get expressed because of different or changed genetic environment. Further investigation is needed why the presence of *bla_OXA-72_* does not produce β-lactam resistance.

MLST is an unambiguous and utility technique that identifies accurate and portable nucleotide sequences of internal fragments from multiple loci housekeeping genes and was used to assess the genetic background of populations in an ocean of bacteria (Maiden et al., 1998). Based on our existing epidemiological data and the eBURST arithmetic, it was shown that CC92 was widespread and this major clone was resistant to carbapenem antibiotics in our hospital. Ruan et al. (2013) explained that CC92 is a widespread variant that has advantages with respect to acquiring resistance determinants and surviving in the nosocomial environment, which renders it preferentially selected under antibiotic pressure. This may be the primary reason for the current epidemiological situation of CC92 in our study. In addition, 82.35% of CRAB from the five predominant STs in CC92 carried the *bla*_OXA-23_ gene. We propose that the *bla*_OXA-23_ gene is tied to the genetic background of CC92, representing an important setting in which to study the emergence of carbapenem resistance. A novel SLVs of CC92, ST843 was identified in three isolates, and sharing six same alleles with ST92 and n1 (**Figure 1A**). We could infer that it may be one of the evolutions of *A. baumannii* to adapt nosocomial environment.

We used eBURST to analyze the distribution of the STs of CSAB, and found it was more dispersive than the distribution of CRAB (**Figure 1**). This may confirm that CSAB has a more diverse genetic background compared to CRAB. The high genetic diversity in CSAB might be attributed to the decreased survival rate of antibiotic treatment. None of them emerge more than once among 35 carbapenem-susceptible isolates from May 2012 to October 2013 except for 3 isolates belonging to CC92. These data also imply that CSAB may have a more diverse genetic source compared to the CRAB, and suggest CC92 is an evolution for *A. baumannii* to survive while antibiotics existed.

We also observed that a few *A. baumannii* isolates could acquire azide resistance after the bacteria were mixed and incubated with *E. coli* J53 together overnight. This can be totally expected since spontaneous mutation(s) conferring azide resistance could arise and be selected in the presence of azide, similar to those *in vitro* selections of drug-resistant mutants. This can be considered as a process of adaptation to a selection pressure. Indeed, mutations conferring resistance to azide have been reported in both *E. coli* (Fortin et al., 1990) and *Acinetobacter* spp. (Elkeles et al., 1994).

The cryptic plasmid pAB52 from CRAB isolate no. 52 does not carry any carbapenem resistance genes. It matches best with pAC12 which was reported in 2012 in Malaysia (Gan et al., 2012), and the ST of this pAB52 CRAB is ST195 (1-3-3-2-2-96-3) in parallel with *A. baumannii* AC12. According to the results of pAB52 sequence blast in NCBI (date not shown), all plasmids carry the same genes with over 99.9% identity over the full length, and belong to the *A. baumannii* plasmid group (GenBank accession number CP008850, CP007535, CP007550, CP001183, CP007578, KJ586856, KJ477078, and CP006964). Thus, plasmid pAB52 is larger with an additional segment of TonB gene (**Figure 2B**), but these plasmids may have the same function. For example, the septicolysin-like protein encoded in these plasmids suggests that it may be involved in survival of bacteria in the lungs and blood (Acosta et al., 2011). The TA systems have been suggested to mediate bacterial persistence by generating slowly growing cells tolerant to antibiotics and environmental changes (Jurenaite et al., 2013). In particular, the pAB52-containing strain was isolated from the sputum of a 74-year-old male who lived in a rural district in western China and had not traveled to any areas before admission to the hospital’s ICU. This individual was found to have acquired an XDR *A. baumannii* infection during his hospital stay and eventually, he died from respiratory failure. The observation suggests that probably plasmid-borne *A. baumannii* TA systems contribute for the evolution of antibiotic resistance in this opportunistic pathogen.

In summary, this investigation confirms that the CC92 producing *bla*_OXA-23_ was the leading reason for the dramatic increase in the carbapenem resistance rates in China. Awareness of carbapenem-resistant organisms and their development in hospitals has crucial implications in optimizing infection control practices, establishing antimicrobial stewardship programs within the hospital, and finally establishing active regional surveillance systems (Zowawi et al., 2015).

## Conflict of Interest Statement

The authors declare that the research was conducted in the absence of any commercial or financial relationships that could be construed as a potential conflict of interest.
