# Supplementary material for: Characterization of carbapenem-resistant Acinetobacter baumannii isolates in a Chinese teaching hospital
Source: Front Microbiol. 2015 Sep 1;6:910. doi: 10.3389/fmicb.2015.00910 (PMC4555021; doi:10.3389/fmicb.2015.00910)
Supplement: Supplementary file 1 [file Table_1.DOCX]

The sequence of pAB52 plasmid

1 gtcggcatta ttctgtagac tttaatacgt tttccttcct gcccacgaat aataggagta

61 gatgctccac ctccatattg attagaatga attcctaatt ccgtacccaa ggcatcccct

121 agagttgtcg aacgctgttt aagtttacta gtatcaataa actgattact aacatttaga

181 cttttgccta acagctcaga ccctaactca tcagattgtg cagttaatac tattgtattt

241 aaggtagtaa caggcccttc agctgccaat aaaattttcg aggcccctaa agtaattaaa

301 gctaaaaata gtttactttt atttagcata aaacaaccaa atttaatgtt atattataat

361 ataacactta aaaatttata aaatctacta tttaaatttt tgaatggtaa tcccccctaa

421 aaatgaagtc aaaaatggga gcctaagctc ccattcttat tgatgatttt ttaattcttt

481 tagcatagca tctcgtaaaa tttcattcat acgtgtttga taacctttgc cttgtgcttt

541 gaaccaagca agtacatcag catctaaccg aattgaagtt tgttgcttta ctgggcggta

601 aaattgattt tggcgtacag cattgctcca gtctgtaatt tcaggaatat ctgatagatc

661 tagctgatca tcaggaaccg tgcctttagc aagcaagcgt tgaatttcag catcttgctt

721 ctcaccaaat ttctcattca gctctttgtg tgagtatcta accatgctca tatttgtttc

781 gctccgcttt agtgacttgc cttgcactaa tgattcggat gatttcacag tcatcttcat

841 caaaaatggt gtgagccacc aataacatta gtacgccttt aactcgtcca atggtttgcc

901 aacgttcttc accattggta tgtctgtctt ggattgagat ccgtaatgga tcttcaaaaa

961 caaggcttgc agtttcgaaa gagatatcat gctttttctg attctttcga ttcttagcct

1021 catcccattc aaaatactgt tccataaaaa acattcaaat ttgtatatac aataatgtat

1081 cacaaaattg tatcactcaa ccagattcgt gtaattttta gtcaaaatta ttggcaagaa

1141 gtatcaaaac tgctcataaa aaagccgact tgtttcaaag tcggcttttt gggcattttg

1201 ggatttttct atttttatta agtagttgat atttatatta atatacaagt gtacttcgta

1261 taatcgccat tatgttaaat agagataaga catactctaa ttttagagtt ttcttttagg

1321 tatgatataa aaatatacaa gcctaacagt ttagtttaaa ggttaaaaat tgaataaatt

1381 aacaaatatt tttatggctt taggagtttg cctttcaatg gaaacatttg cagaacctgt

1441 tttttctaat gatttattag cgaaagcaga aaatggagat acttctgctc agttagaatt

1501 agccgagatt tatctatatg gtcatggtgt tgattcagat gaaaatcaag ctgaagtttg

1561 ggctcttaaa tcagcagaaa atggaaatgt agcagcaatg ttttggttag ctgatggata

1621 tgttacttat gctagattaa tggaagatga tgacaaaaac gattctttag aacatttcca

1681 aaaagctttt aagtggtttc agaaagcctc agaaaatggc cattctgaat caatggttga

1741 gttagctgat ctatatactc gtgcagatag cggaatagaa gttaatatta ataaagcttt

1801 tgaacttcgt gaaaaggctg caaagttagg taataagaaa gcaatgcgaa gtctttccgt

1861 tatgtatcgt gatggtatag gtattcctaa aaatactgat ttagctcaaa gttggtggga

1921 taagtctgaa aattagtttt aatatttcct aaaattaaca taatacacct tatacgaaat

1981 gctgtataat tatccttata attcaacgct gtagtttcta gtttatagcc cgagcatccc

2041 tgttcgggct ttttttatga cttttcacgt tccacgctat tatttcatca atgttccaca

2101 cttttgatta gctcaattga ataatcagga tgcctggtct ttttcttttt ggcattcatc

2161 cagctctttc ttcagcgcct ttttatcctg gtacgatgca atcagcatga tcgggatcag

2221 cagcggagct acaataattc ccagggcaat gtctttcagt ttcggcatga tctttcaccc

2281 ttattgctca gtcattatca aagacggcat tcagcacacg gccaaataaa ccacgttttt

2341 tgtgttgtgg cactgccaca ctttgcaacg tggcatgctg tagcgtttgg gtgtggtatg

2401 ccgtagtttc ttgctgtggc actgctacat tttctgcaat atttaattca ttattctttt

2461 tgttatcagt gtcttgttgt gttgtcgcat gctgtggcat cttggtgtcg catgccgtag

2521 catcttggtg tggtgctgct gtggcaggtg caacactttt cagctcaatg agtgtttgca

2581 tacggtcaat ttgttgcatt aaccgttctt cacgttcttg gtattttttg acttcatttt

2641 ctaaatgggc aataagtttt tttgtagatt catcaccttc ttgcttagat gattcaattt

2701 tttgagatgt ttgctcaggt tctccaaagc atcgtatgac ctctgctaga tcgaattgtc

2761 catcactgga gcgtgataaa cgtccattac gtacagctct gtaaatggtt gatctatcca

2821 catgaaattt tttagatact tcacttagtg ataacttagc catttacggt gtccttttta

2881 tttgatggca tgtatcccac tttttttagg tatgggtata gctctttgaa ttgagcggga

2941 tcttgtagca tctcagcaat acgtacagca aactgttggt agctttctgt tccttgtgaa

3001 tagcgaccca tttcagggag ttctgaaagt ttatttgcaa acatatgccg ttgagcatcg

3061 gtcatctttg aaaaaagctc caaagtgttc ggatctcttt ctattgatgg gctatccttc

3121 ttcttctgtt taaaggtaaa agaaaatcct gaaatagaac gtcctctttt atgctgttca

3181 tattttacag ttatatccgt atgctcattt atttgtttaa ttgaaagctc taaaacacgc

3241 tcttttaaat gagccatccg taaatattca ttatcaagaa cgcctaactt ctttctaaat

3301 tcttgtagtt ctatggttgg ggttttacca gtgcttcgcc actgaattag aagctcatat

3361 aagcgaatgg cataagcgct actaagacta ctaacttgct gtaattcata tttagtaaat

3421 tgttcttcca gtcgggttat gaagggaaca acagcaggtg caaatattaa gtctacagtt

3481 gcttcattgt cgttataagc aatttgagat acccaacgac tcataacatt tcggatattc

3541 ccattagctt ttttctcttg atagctaaat tgacgcgcga ataaatcctt acaagcatct

3601 tttaaggctt gataagctgt attacggtgt acaccaaatt gattgatata actatctgca

3661 tgaacttcta atggatcatt tgcattaatc cctttcccac tttcccttgc ttcaacaata

3721 gccaataaaa ttaaacgttg ttctactaag tctaagttat agcttgcgtt gattaaggca

3781 ttgtccttta caactaaatc tctcatagcc actatttttt attgtgtata agtacacgtt

3841 aatattattt gtgtactaat gcaaggtaaa cgtggacata ttgcaaggta aacgtggaca

3901 tattgcaagg taaacgtgga catattgcaa ggtaaacgtg gacatataag agctaaaacc

3961 ctttattcat aaggctttca agccatttaa aagtatttaa aaactttaaa ataattaaaa

4021 gcccagggaa tgaaaaattg cccttggttt tcgcttcgct caaactctat tgaacttcgc

4081 tttcgctcag ttcgttgggg caattttttg gttaatactg gcgtgacttt aaaaaaaagc

4141 aaaagaaact tggaattcgc ttcgctcata aaactttttt actcgctacg ctcgatccag

4201 gagcaaattt ctgcacgaaa cttacgcttt cttaggctaa agaggacaaa aaaatgtttt

4261 tcagagggtc taggcgcaaa ttttgatagt tcgctcgtag acactcgctc tatgcatcta

4321 tctaaagtca aaatacctct ttatttcgct ctcatttgcg ttttttgagc tgttttgtgt

4381 ttttcactat gagatctatg ttttctaggt aaaggctctt aaatcgcatt tgaacgcgtt

4441 ctatgaagct tttcataatt aaagttttgg gttgaaattt ttaacctgga tgaatggaca

4501 aaaaattgat tttttacgtt tttctaaaca aggaaataaa tacttttttc catgcaaaac

4561 caagcaccgg ccctacgtca ctttagccaa ttttccaatc caagatccgg cacatgccgg

4621 gatttttttg tcacacacca cgcatctaac gatgacaccc ctcaaagcct tacaggataa

4681 ggatttcagc gattttttag ggtctcagaa acgtgtacag aaaaatggtc tttttttaga

4741 ataagtgatt gaattatcta ttttttatgc tgtgctcatg gttgtacatc aaaacaacac

4801 tttttttaga taagatattg ataaatttaa gaaaaaatat gtactcatga ttgtacataa

4861 aaaaagcatt atatttaaat aagtttttgt tttagaataa attaattctg tacaaataag

4921 tttgtacgta atctaattat tattttttat ttaattagtt gaattatata tatttaattt

4981 tgtacaaaat ctaattttaa taagaggtgt aatttatggg cgaaaaagtc aaaacttatg

5041 cagtcaggtt agatccacaa gtagcagaat tctacgatca attggctaat tcagaaggaa

5101 tcagaaccag taaattattc agtaaaattt tgaccaatga ttttcaatct attgctgtta

5161 aacagcaagc cgatcgtatt gaggatctgg tgaatcgatt agaaaagcga atagatgatt

5221 ttgaggaaga taacaatttt tctgaaaaat actatgaaga cttttctgga ctatatttca

5281 tgatgttgtg gctcttaatg aaaaatggcg cttctaaaga tgaggttaga atgatacaag

5341 ctaaaggtta ttcatatgca gatgataact ataagagggt aaagtagtgt cagataatat

5401 tattcagctt tcaaagccat gtacgctttg tgaaaataga caaaatgtac aactttttgc

5461 aggattgatg ctgtgtggaa gttgtcagga aaatatcagg cttacgaatc caagtatgtt

5521 ctcggccaat gatgagattg aacaaaaagc ccagaattaa cctgggcttc gtataagaga

5581 ttttatgtta aatgccgaac tgaattaaaa atagaaatgt agttgagctg atgcttgttc

5641 atttctttat ttttagtaat tgaggtgaac atactgtcag tacatggtag ttataagtaa

5701 cgtgtaactt tgtttgcaca aagatttgag ttatgccaag tgatggattg atttgtactt

5761 aaaattttaa gcttatccac ttgctttgca agccactaca ctttatttac aatcctatgt

5821 tgtggaagta agggatcttg tactcgccca gtatgttcaa cttgattata tctaaaggac

5881 tattaggctt caatcaaatc aattttccaa ttagtcgact gaattaacac gtttaaatca

5941 cgtaatttaa gactaatatc atcagcttgt ttttgtaatg atgaaactgg aataacttta

6001 tgccacttta tttcccgcgt actataacga tcagattcac gagatgtatt acttattgct

6061 gcagttaggg ttttgtgctg ttctacaaat tcatcacgca aagtcaataa agcaagtaga

6121 gaacggccat catttaattt tgttaaagca ttagtttcat gaattgcata acttaatttt

6181 tgaaattctt gaattaaaga aaatacttgt tttagtagtt catttggatc ttcagatggt

6241 tgatcaccat cttgtaccaa tacatttgca ttgatacgtt gttttagtga aataattttt

6301 ttttgttgat cacttcgaag taagagagct tctgctaatt tcatttttca cctattaacc

6361 aattagaatt aagttttaaa cagcttatat cctcattctt taaagatcaa aaaaattgct

6421 ttttatctag ctaaaaggtg tctacaaaat cggtggctat tcagtgctga aaaggctgcc

6481 gacacataaa gtgacccaga ttgaagagtt actgccacac tgctggaaac ctaaatcgaa

6541 ttaaaaaata ggtatgggat tcagcggacg cttaccttaa ttattaatct aatcatttta

6601 taagaaaaat cttctaagaa attttcttag aagattacag tatcttatct agaaattaag

6661 atttaagcct aaactaaaat ttcttccaat ttggggaata tatggcaaaa atgtttcatg

6721 ggcatagact ttctgatcaa gtaaattatt cgctttaaag aataaatcat attctgtgtg

6781 agatagctta tttttatatg ctaaagtcat attaagcata ttataacctg atgtaacctg

6841 ctcaaattta gaaatattat cttgtttaaa gactcgataa tattctattt cacctttaag

6901 cgattcatca aaatcagctt ttagtcgtgt acccaaacga gcaggaggta atctaggagt

6961 gtaacgatcc ttttggggag ctaaagtcgg ttctctattc cagatatcgt atgcaattac

7021 agcatcaggt aaatttgtta aacggccttt cacataatct ccaaaaagag agccgtgata

7081 aacagaatta aattgatacc ctatgttgcc ctctaatcca taaaaattag cagcagactg

7141 gctataatga ttaatacgta gtgtatgctg atctctaact tttgtagttc ctaaaacttc

7201 attgagtgtc tgtaaatata tatagttatc aaaatcataa tagtaagtac tgatttgata

7261 atcaagtaaa tcatccttat aggccagact gatttctaaa ttattggatt tctcttttct

7321 tagaaagcgg ttacctattt caaaagaatt agtagcgaga tgcataccat gagtatacat

7381 ttcttgtgca ttaggtaatc tttcttgatg tgtacctgtt aatgataaga tataatttgg

7441 agcaaatctc cagtggactg tacctgcata agaaaaagct gtttctttgt taggttgaac

7501 agcttctaaa atagatttta aattttgagc cctgatttta ttattttttt ctacataagg

7561 actgttatat ttattaggcc aaggtttcat tgaatcttta attttttcta tatcataatc

7621 catactaact ttctgctttt ctatacgagc acctagttcg aaagtaaagt cgttccagtt

7681 atatttttct aggccaaata cactaaagtt tttagtgaca tttcgattta atagctgctg

7741 attatctaga taggtggtat ggccttcttg agaatggact ggtgataaag cactattatc

7801 ctgttccaaa tattgtagcc ccaagatacc ggttaattcc cctaatggct gatgtgataa

7861 ctctatgcgg cctacttttc ctgtattttt aaaaaagtta gtaatcttat ctccctcgag

7921 ttcattgtga aaataatcta tataactcaa tgaggtacga attttgtcaa taccggtaaa

7981 tggttgagtg aattctccac gcacatcata acgtcgagta tttaaatcaa tataaggagc

8041 attatgagag aaattagttt catgaatatg attatgctga tgacaatcag gacgtggatt

8101 tatgtaatta atatcttgct catccatcaa ttgaggataa tataaaagat aattttttaa

8161 tccactaatt cgtgaatcga taccgattgc agaggcacca caaccttcat ataaatggct

8221 atgggctggt aaaccatatt catcatgacg atgtgtataa gaaactccta aataagtatt

8281 atcgtctatc caggataatc caagcgtacc tactctcgac tctgcccagc tttctggtaa

8341 atattttaaa tttttataaa tactgttttg gttttcaaga tcttctaaag aattataaga

8401 gctagactgg taatgaggag ttttatagtt accagcatta cgatataacc cttctgcccg

8461 taaagctaca tgtgggctta agccaactgt tacacctgct gtggtcaact tctcattact

8521 gccagtatta aaacgtaatg tagtattacc ctctaatccg tcttgaggca tttgggttgg

8581 aattttatag tcaatgacat tgactacccc tgccgcacta tttgaactat acaaaagggt

8641 acttgcacca cgtataattt caatagactt agctaaactt ggttcaactg tgacagcgtg

8701 atctggggac atattagaca tatccaacac gtcagcatta ttctgtaaaa ctttaatacg

8761 ttttccttcc tgcccacgaa taataggagt agatgctcca cctccatatt gattagaatg

8821 aattcctaat tccgtaccca aggcatcccc tagagttgtc gaacgctgtt taagtttact

8881 agtatcaata aac
